# Epidemiological Methods: About Time

**DOI:** 10.3390/ijerph7010029

**Published:** 2009-12-31

**Authors:** Helena Chmura Kraemer

**Affiliations:** 1 Department of Psychiatry and Behavioral Sciences, Stanford University, 1116 Forest Avenue, Palo Alto, CA 94301, USA; 2 Department of Psychiatry, University of Pittsburgh, 3811 O’Hara Street, Pittsburgh, PA 15213, USA; E-Mail: hckhome@pacbell.net; Tel.: +1-650-328-7564

**Keywords:** risk factors, statistical and clinical significance, effect sizes, moderators, mediators

## Abstract

Epidemiological studies often produce false positive results due to use of statistical approaches that either ignore or distort time. The three time-related issues of focus in this discussion are: (1) cross-sectional *vs.* cohort studies, (2) statistical significance *vs.* public health significance, and (3), how risk factors “work together” to impact public health significance. The issue of time should be central to all thinking in epidemiology research, affecting sampling, measurement, design, analysis and, perhaps most important, the interpretation of results that might influence clinical and public-health decision-making and subsequent clinical research.

## 1. Introduction

Epidemiology has been defined as the “study of the distribution and determinants of health-related states or events in specified populations, and the application of this study to control of health problems” [1] (Page 55). “Distribution” refers to incidence or prevalence of disorders *in specified time periods.* “Determinants” refers to risk factors (some causal, some not) for disorders, factors that can be shown to identify individuals who *at a later time* are more likely to have the disorder. “Application” refers in part to prevention of disorders in the *subsequent period of time* by manipulating causal risk factors among those as yet without the disorder. All such tasks are crucially dependent on considerations of time. While other types of questions also fall within the bailiwick of epidemiology research, the unique contribution of epidemiology research is guidance based on observational studies provided for the prediction and prevention of disorders, both crucially time-related. Yet such epidemiological studies have often produced false positive results [2–5], even studies that seem well and carefully designed, executed, and analyzed. At least part of that problem relates to the statistical methods used in epidemiology, many of which ignore or distort time.

In what follows, we address three interlocking areas of such concern in observational studies in human populations: (1) cross-sectional *vs.* prospective longitudinal (cohort) studies and their design; (2) statistical significance *vs.* public health significance and (3), how risk factors “work together” to impact public health significance. None of these issues is completely novel, but the problems they generate continue to affect epidemiological studies, and it is worth considering why this is so.

## 2. Cross-Sectional and Prospective Longitudinal (Cohort) Studies

To show that some factor is a “risk factor” for a disorder, it must be shown both that: (a) the factor precedes onset of the disorder, and (b) it is correlated with the disorder [6]. A factor that is correlated with presence/absence of a disorder in a cross-sectional study may be a symptom of, or a result of, the disorder, not a risk factor for it. Presence or absence of the disorder may be indicated either by prevalence or incidence within a specified time period.

For a disorder that is chronic and persistent after onset, prevalence at a certain age equals the incidence between birth and that age for those that survive to that age. Otherwise, for a disorder from which there may be remissions or recoveries, or one associated with removals from the population, cross sectional correlations may relate as much to the association of factors related to treatment availability and response, or inconsistency of expression over time, as to risk of incurring the disorder. Consequently cross-sectional studies investigating correlates of prevalence of episodic disorders are of limited use in identifying determinants of those disorders or ways of preventing them, although they may be vital in setting the stage for prospective studies to accomplish those purposes.

One exception relates to the detection of “fixed markers” [6], *i.e.*, risk factors that are fixed over the course of the lifetime of individuals, e.g., gender, ethnicity/race, year of birth, and, in general, genotype. Since fixed markers exist at birth and remain unchanged over the lifetime of the individual, these are temporally precedent to onset of all disorders during the individual’s lifetime. Thus to show correlation between a fixed marker and presence/absence of a disorder in a cross-sectional study is to establish that the variable is a risk factor for that disorder, although the association may be weaker than that between the fixed marker and an incidence. Fixed markers are crucial to identification of “high risk” subpopulations, thus both to designing research studies and to targeting interventions, but since fixed markers cannot be changed, they are of little value to designing interventions to prevent onset of disorders.

**Figure 1.**
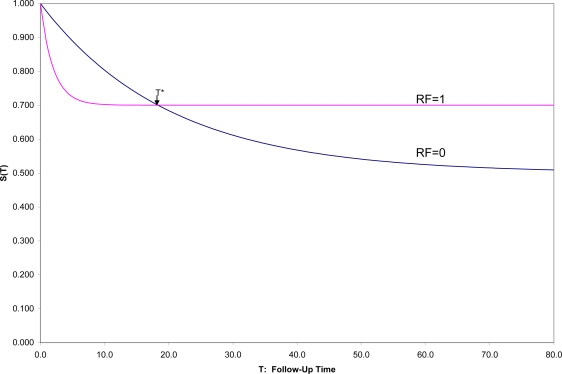
Comparison of two hypothetical survival curves for the subpopulations with RF = 1 and RF = 0 (non-proportional hazards).

The ideal, but admittedly unrealistic, approach to demonstrate risk factor status would be to sample the relevant population disorder free at a designated time zero (t = 0), to evaluate potential risk factors at that time, to follow each sampled individual over his/her subsequent lifetime, in order to evaluate and compare survival curves to the onset of the disorder [7] over that time period to see for whom and when onsets occur. With a binary risk factor (RF = 1 identifying “high risk” individuals and RF = 0 “low-risk”), one might see a situation such as that in Figure 1, which shows hypothetical survival curves to onset of a disorder for the “high risk” subpopulation (S_1_(t) in those with RF = 1) and the “low risk” subpopulation (S_0_(t) in those with RF = 0) for all values of t.

Then one could not only compare the overall survival curves, but could compare the incidence between time 0 and any fixed time T [1-S_1_(T) *vs.* 1-S_0_(T)]. In this hypothetical example, (1) the survival curves cross at t = T*, an unusual, although not an unknown, situation, and (2) onset is not inevitable for every individual, which is a quite common situation. In this example, 50% in one group have onset during their lifetimes compared to 30% in the other group, but the latter are likely to have their onset early if at all, which results in the crossing of survival curves, here at T* ≈ 18.

Here if incidences prior to T* are compared, RF would appropriately be described as a *risk factor for* the disorder; after T*, as a *protective factor against* the disorder. Exactly at T* the factor is unrelated to the incidence of the disorder. This illustrates the general principle that conclusions concerning the relationship of any risk factor for a disorder may depend strongly on the exact time span to which incidence refers.

Because it is more convenient to have short follow-up times, epidemiologists often assume that whatever the relative positions of the two survival curves near zero are the relative positions for all follow-up times. This is one of those extrapolations that often mislead both subsequent research efforts and clinical decision making. In general, the inferences from a cohort study apply to those in the disorder-free subpopulation represented in the sample at time 0, and followed for as long as the study chooses to follow the individuals, whether that be 1, 2, 5 or 10 years, but not necessarily to another population, and for any longer. Explicit presentation of the estimated survival curves up to the end of follow-up, as a general practice, would not only inform medical consumers as to exactly the findings of the study up to the duration of follow-up, but also remind them of the time limitations on inferences.

Standard survival methods [8] used to estimate survival curves deal well with “censored” data. Thus if the intended follow-up time is 10 years, but some individuals are lost to follow-up, still onset-free, after 1,2,3,… years, the survival curves can still be estimated up to 10 years using these methods, *provided* loss to follow-up is not associated with the same mechanism that produces onset. Thus, for example, in comparing those who did or did not use hormone replacement therapy (HRT) on time to onset of coronary disease [9], one cannot treat those with onset of cancer, cognitive disability, or the occurrence of a stroke as a “censored” data point, for HRT may be a risk factor for all these outcomes [10]. If such competing outcomes were mutually exclusive, observing the occurrence of any one before the others would indicate non-occurrence of the others at any later time, in which case that would not be a “censored” data point, but a signal of the longest possible survival time. However, such outcomes are not usually mutually exclusive. One may, for example, have both onset of coronary disease and subsequently onset of cognitive disability or *vice versa*. This is an unavoidable problem when risk factors are, as they often are, non-specific to one disorder, and epidemiologists seek to estimate the effect on each specific disorder separately.

Moreover, how the zero-point of time (t = 0) is defined makes a major difference in conclusions. For example, in the hypothetical situation in Figure 1, let us say that t = 0 refers to time of birth for the individuals in the population. If instead, individuals were sampled at the age of 10 or 20 or 50 (t = 0 in each case defined as the age at which individuals are sampled and their risk factors assessed), the populations sampled would differ, for those still disorder-free at age 10 are a non-random subset of those still disorder-free at age 20, *etc.* Moreover, any risk factor not a fixed marker might change within individuals from age 10 to 20 to 50, and the risk factor measured at different entry ages may have a different association with subsequent onset (age may moderate the effect of the risk factor on outcome). In general, the survival curves for those entering at different ages would be quite different from each other, and the conclusions might differ as well.

More problematic is the situation in which disorder-free individuals over a wide span of ages, say 20–80 years, are sampled, and t = 0 refers to the more or less arbitrary time each individual enters the research study. Time of entry to an observational research study (here the focus) has no clinical relevance to the individuals in the population to which inferences are to be drawn. In that case, the observed survival curve is a mixture of the survival curves of those who enter at each age disorder-free with the risk factors as they are at that age, the mixture determined by the age distribution in the samples. Different studies are unlikely to reproduce and confirm the same findings, particularly when both the factor (e.g., use of HRT) and the disorder itself (e.g., heart disease, diabetes, cancer, Alzheimer’s disease) are age-related.

Again, there is one rare situation that is an exception to these concerns: the constant hazards situation with fixed markers. If the risk factor is constant over the lifetime of the individual and the probability of survival for any time span is exactly the same regardless of when a individual enters a study (exponential survival curve, Poisson distribution of events), it doesn’t matter at what times the individuals are sampled, or whether they are sampled at the same time or what the distribution of entry times in the high- and low- risk subpopulations were. It doesn’t even matter how long individuals are followed or why they drop out. This is also the one and only case in which the incidence rate (“events per person-year”) estimates an interpretable population parameter, namely the reciprocal of the mean time to event, regardless of entry and exit times [11].

However, not only are many risk factors of interest not fixed (e.g., HRT use), there are few, if any, real onset distributions that follow a constant hazards model. Only an inevitable outcome (e.g., death) can possibly follow a constant hazards model. No age-related disorder (e.g., heart disease, cancer, Alzheimer’s disease or even death) can. Nevertheless epidemiological studies still occasionally use the incidence rate to compare the high- and low-risk subgroups [9,12] as well as other methods that depend on an unacknowledged assumption of constant hazards. Such studies have a high chance of drawing misleading conclusions [3].

In a prospective observational study, with a representative sample from the disorder-free population of interest, all entered at a *relevant* zero time (fixed short age span or a fixed life event) and followed for a reasonable *fixed* period of time, the association between a risk factor and onset is due *either* to a causal effect of the risk factor, *and*/*or* to any factor(s) associated with that risk factor. Attribution of causality to risk factors detected in observational studies must be tentative. In what follows, because the focus is on observational studies, no causal inferences will be drawn. Inferences apply only to the population sampled free of the disorder at a relevant zero time. For clarity of communication, all risk factors considered here are binary (RF = 1 *vs.* RF = 0), although the principles apply more generally. Finally, the outcome of interest is incidence between time zero at which time RF is measured, and a fixed time T for all individuals, the object to compare 1-S_1_(T) *vs.* 1-S_0_(T).

## 3. Statistical Significance is NOT the Goal: Public Health Significance Is

Over the last 20 years, considerable attention has been paid to the overuse, misuse, abuse of “statistical significance” [13–21]. In the way statistical hypothesis testing is usually used, to say an association between a risk factor and subsequent onset of disorder is statistically significant is at best to say that the sample was large enough to detect a non-random effect. Such a non-random effect may be of trivial public health significance. What is usually reported is the “p-value”, a statistic computed with the study data. A level of significance is set ‘*a priori*’, α (typically 0.05). If p < α, then the result is said to be “statistically significant” at the α level of significance.

However, unless the null hypothesis is absolutely true, the expected value of the p-value approaches zero as the sample size increases, rapidly for a strong effect, slowly for an effect of trivial public health significance. Meehl and others [22,23] point out that the null hypothesis of randomness is never *absolutely* true. If so, no matter how trivial the non-random association between risk factor and outcome, it will be found “statistically significant” at whatever significance level is specified, provided only that the sample size is large enough. Consequently, the p-value serves more as an indicator of adequacy of the sample size to detect whatever effect size actually exists, than as an indicator of public health significance. A reviewer suggests, however, that there is a danger in ignoring the chance element in determining the p-value, that interpreting the p-value as equivalent to “adequate power” could lead to unacceptable publication bias. To show public health significance, an interpretable effect size is necessary, a population parameter that reflects the strength of association between the risk factor and the onset for public health purposes. There are a number of viable such effect sizes.

For example:

- AUC [24–29]is the probability that a low-risk individual will have better outcome than a high-risk one, where ties are broken with a toss of a fair coin: here AUC = .5(S_0_(T) − S_1_(T) + 1).
- Success Rate Difference (SRD) [25,30] is the difference between the probability that a low-risk individual will have better outcome than a high-risk one and the probability that a high-risk individual will have a better outcome than a low-risk one: here SRD = S_0_(T) − S_1_(T) = 2AUC − 1 (in epidemiology, SRD is usually called the risk difference).
- Number Needed to Take (NNT) [25,31–38] equals 1/SRD or 1/(2AUC - 1). For a binary risk factor and incidence between 0 and T, NNT is the number of high risk individuals one would have to take at time 0 to find one more onset prior to T than if the same number of low risk individuals had been taken.

While these are three mathematically equivalent effect sizes, generally NNT is easier to interpret in terms of public health significance, and SRD and AUC are easier used in computations (e.g., for confidence intervals).

There are, of course, other viable effect sizes applicable in special circumstances. For example, Cohen’s d [39] is appropriate when the survival times in the high- and low- risk groups are normally distributed (an unusual situation): d = (μ_1_ − μ_0_)/σ, where μ_1_ and μ_0_ are the two group mean times of onset, and σ^2^ is the average of the two group variances. Then SRD = 2Φ(d/√2) − 1, where Φ() is the cumulative standard normal distribution. In the rare situation in which the constant hazards model holds in both groups, SRD = (μ_1_ − μ_2_)/(μ_1_ + μ_2_) = (RR − 1)/(RR + 1), where RR, a relative risk, is the ratio of the incidence rates in the two groups. There is limited applicability of such specialized effect sizes, but, when applicable, they are easily converted to SRD (NNT, AUC).

While the expected value of the p-value approaches zero as sample size increases, the sample estimate of an effect size, e.g., SRD, estimates the same population parameter regardless of the sample size. Instead, as sample size increases, the width of its confidence interval decreases to zero, *i.e.*, large sample size improves the accuracy of effect size estimation, and does not change the effect size estimated.

A question that deserves careful future consideration is which values of NNT indicate public health significance, and which are trivial. For example, one would question any recommendation for costly and risky surgery on 500 patients to prevent one onset of coronary disease, *i.e.*, to subject 499 patients unnecessarily to costly and risky surgery to prevent one onset. On the other hand, one might well be willing to recommend half a baby aspirin a day to 500 patients to prevent one such onset. In short, there is no universal answer to this question—the answer depends on how serious the disorder is, the consequences of untreated disorder, whether effective preventive intervention is available, how costly and risky such intervention might be, the vulnerability of the population and other such considerations. With the focus to date on “statistically significant” results, such questions have yet to be discussed seriously. Instead larger and larger sample sizes are used to detect ever smaller effect sizes, many of trivial public-health significance.

Epidemiologists often use the Odds Ratio (OR) as such an effect size, but OR is not viable in this role. Historically, OR was introduced as the likelihood-ratio test statistic to test the null hypothesis of randomness. OR remains useful as a detector of non-randomness, for example, in logistic regression analysis models: OR equal to 1 indicates random association; greater than 1, positive association, and less than 1, negative association. However, there is no magnitude of Odds Ratio unequal to 1 that unequivocally indicates public health significance.

**Figure 2.**
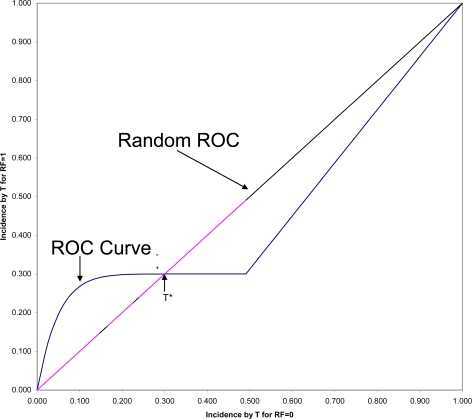
ROC curve comparing survival in the “high” and “low” risk groups.

Many arguments have been put forward in recent years to support that contentious point [40–44], but let us here consider only one closely related to the consideration of time. In Figure 2 is shown the ROC (Receiver Operating Characteristic) plane in which the two survival curves shown in Figure 1 are compared. Here 1-S_1_(t) is graphed against 1-S_0_(t) for all values of t. These values connected with each other and with the two endpoints at (0,0) and (1,1) form the ROC curve comparing the RF = 1 and RF = 0 groups on time to onset (AUC is the area under this ROC curve). If there were only random association between the risk factor and onset, the ROC curve would coincide with the diagonal line from (0,0) to (1,1): the Random ROC. Here the ROC curve clearly indicates non-random association. The ROC curve crosses the Random ROC when t = T*, and as t increases, all points converge to the single point (0.5,0.3) determined by the proportion of the two groups who will eventually have this non-inevitable onset.

For any fixed follow-up time, T, there is one point on the ROC curve (1-S_1_(T), 1-S_0_(T)) that indicates the strength of association between the risk factor and that particular incidence. The SRD for such a binary outcome is proportional to the distance between that point and the Random ROC[42]. Since all ROC curves begin in the lower corner of the *ROC* plane, it is clear that the SRD measuring the association between the risk factor and any incidence depends strongly on T, and will always approach SRD = 0 as T approaches zero. However, Odds Ratio often tends to go in the opposite direction, becoming very large for very short follow-up periods, in some cases even approaching infinity as T approaches zero.

**Figure 3.**
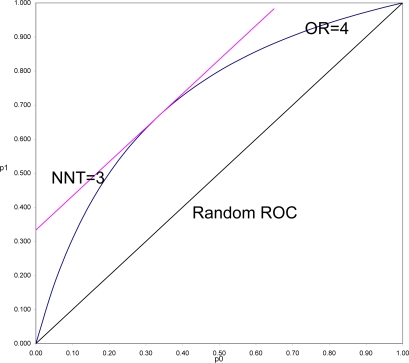
Equipotency curves comparing the locus of all points (p_0_, p_1_) with p_1_ > p_0_, the ROC plane with Odds Ratio = p_1_(1 − p_0_)/[(1 − p_1_)p_0_] and NNT = 1/(p_1_ − p_0_) = 3. (The Figure 3 here selected to be equal to (OR^1/2^ + 1)/(OR^1/2^ − 1)).

To demonstrate this and to understand why this is often so, it is necessary to put OR and SRD on comparable scales. In Figure 3 is shown the location of all pairs of probabilities (p_1_, p_0_) that would yield OR = 4 (OR = p_1_(1 − p_0_)/((1 − p_1_)p_0_)), and all pairs of probabilities that would yield SRD = 1/3 or NNT = 1/SRD = 3 (equipotency curves[43]). The same demonstration could be done for any fixed value of OR > 1, with SRD = (OR^1/2^ − 1)/(OR^1/2^ + 1) = Y (Yule’s Index).

What is in Figure 3 seen is generally true. SRD is constant (here equal 3) for all pairs of probabilities a fixed distance above the Random ROC. On the other hand, OR is constant (here equal 4) for all values on a curve symmetric around the line p_1_ = 1 − p_0_, and beginning and ending at the points of the Random ROC at (0,0) and (1,1). For fixed OR > 1, the *maximal* distance from the Random ROC coincides with the *constant* distance for SRD = (OR^1/2^ − 1)/(OR^1/2^ + 1). That distance then decreases to zero at both corners of the ROC plane. Thus it is always true that for positive association, SRD ≤ (OR^1/2^ − 1)/(OR^1/2^ + 1). In the special case when p_1_ = 1 − p_0_, the SRD = Y = (OR^1/2^ − 1)/(OR^1/2^ + 1). When p_0_ and p_1_ are both of moderate size (say between 0.25 and 0.75) then SRD is approximately equal to Y = (OR^1/2^ − 1)/(OR^1/2^ + 1).

In a ROC comparing survival curves as in Figure 3, to be informed, for example, that NNT = 3 (SRD = 1/3) is to be assured that the location of the point (1 − S_1_(T), 1 − S_0_(T)) is bounded away from random association. To be informed that OR = 4 allows the possibility of being as far away from random as is NNT = 3, but also allows the possibility of being arbitrarily close to random association, particularly when T is small.

In the comparison of any two survival curves, points corresponding to very short follow-up times are always near the lower left corner of the ROC plane, and very near the Random ROC. These points will have SRD = 1/NNT near zero indicating weak association, but since all the OR > 1 equipotency curves converge in that corner, these points often have very large OR. Simply stated, the problem is that the denominator of Odds Ratio approaches zero as T approaches zero, and division by zero tends both to “explode” the magnitude of any ratio and to make it very unstable. For this reason and all its many consequences, Odds Ratio should continue to be used as an indicator of non-randomness, to test null hypotheses of randomness, but not to be used as an effect size.

**Figure 4.**
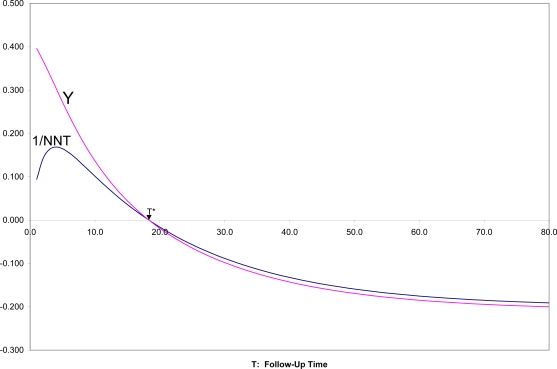
Comparison of Y = (OR^1/2^ − 1)/(OR^1/2^ + 1) with 1/NNT for various follow-up times for the survival curves shown in Figure 1.

In Figure 4 are shown SRD = 1/NNT and Y = (OR^1/2^ − 1)/(OR^1/2^ + 1) for all follow-up times T, for the two survival curves in Figures 1,2. It can there be seen that for T near zero, OR is very large (Y = 0.4 corresponds to OR = 5.4), but SRD is near zero (NNT approaching infinity). For t = T*, as appropriate, both OR = 1 and SRD = 0 indicate random association. For t > T*, when both survival probabilities are in the middle range between 0.25 and 0.75, SRD is approximately equal to Y = (OR^1/2^ − 1)/(OR^1/2^ + 1).

## 4. How Risk Factors “Work Together”

Since the common use of Odds Ratio as if it were an effect size tends to exaggerate the association between risk factors and disorders, moving to use of SRD, NNT or AUC instead will only diminish the apparent clinical importance of many risk factors. This is unwelcome news, but probably reflective of the truth. A few special cases such as infectious diseases or single gene disorders aside, there are probably very few disorders for which a single risk factor can completely explain onset. It is likely that for complex disorders (heart disease, cancer, psychiatric disorders) multiple risk factors “work together” in parallel or in sequence to have influence the onset of a disorder. Thus examining how risk factors “work together” is crucial to prediction and prevention efforts.

To date, multiple possible risk factors are often simply included as independent variables in a linear model, completely ignoring (1) their timing relative to each other, (2) possible correlations between risk factors, (3) their possible interactive effects on incidence. Moreover the linearity assumptions and the link function selected (e.g., log-*odds*) often do not well fit the population.

The MacArthur Model is an alternative approach that takes these factors into consideration [45–49]. The distribution of two binary risk factors RF_1_ and RF_2_ in the population of interest is shown in Table 1. In that population the probabilities that RF_1_ = 1 is Q, and that RF_2_ = 1 is P. The parameter ρ (the product moment correlation or phi coefficient between the risk factors) is an indicator of non-random association between the two risk factors in that population, with ρ = 0 indicating stochastic independence between them.

In Table 2 are shown the incidences between time 0 and fixed T, in each of the four subgroups defined by the two risk factors. When using SRDs as the effect size, the conditional SRDs for RF_1_ for the two values of RF_2_, and the conditional SRDs for RF_2_ for the two values of RF_1_ are also shown. The “main effect of RF_1_” (ME_1_), and the “main effect of RF_2_” (ME_2_) are respectively the averages of the corresponding conditional SRDs, and the “interaction effect of RF_1_ and RF_2_” (INT) is the difference between those conditional SRDs (the same for both sets of conditional SRDs).

If RF_2_ were ignored, the SRD relating RF_1_ to outcome (for each possible value of T) is equal to:

SRD1=ME1+ρ(PP'/QQ')1/2ME2+.5*INT[(2P−1)−ρ(PP'/QQ')1/2(2Q−1)].

If RF_1_ were ignored, the SRD relating RF_2_ to outcome is equal to:

SRD2=ME2+ρ(QQ'/PP')1/2ME1+.5*INT[(2Q−1)−ρ(QQ'/PP')1/2(2P−1)].

SRD_1_ and SRD_2_ are called the “raw”, “overall”, “marginal”, or “univariate” effects of RF_1_ and RF_2_ on the outcome, indicating the association of that risk factor on outcome in the population sampled when all other risk factors are ignored. While the formulas above are exact only for two binary risk factors predicting incidence between 0 and fixed T, the principle is true in general. The “raw” effect of a risk factor (SRD_1_ or SRD_2_) comprises three sources: the unique effect of the risk factor itself, the main effects of other risk factors correlated with the risk factor of interest, and the interactions of the risk factor of interest with other risk factors. How much of each source is represented depends on the joint distributions of the risk factors in the population (here P and Q and their correlation as indicated in Table 1).

The main effect of a risk factor of interest (ME_1_ or ME_2_) does not convey the effect size in the total population unless the other risk factor(s) are neither correlated (ρ = 0) nor interactive (INT = 0) for the risk factor of interest. Nor does the main effect of a risk factor convey the strength of association in each subpopulation “matched” on other risk factor, unless there is no interactive effect. In short, the research questions addressed by the raw effect size SRD_1_, the conditional SRDs for risk factor 1 in the subpopulations with RF_2_ = 1 and 0, and the main effect of RF_1_, are all usually different, not because one is “right” and the others “wrong”, but because they address the association of RF_1_ with outcome in different populations. Thus “adjusting” for RF_2_ in a linear model does not usually “remove the effect of RF_2_”. It changes the research question from that of focusing on the effect size of RF_1_ in the total population sampled, to that of the common effect size of RF_1_ in the subpopulations defined by RF_2_ in absence of an interaction, or to some weighting of those effect sizes in the presence of an interaction, the weighting determined by whether or not the interaction was included in the linear model and the joint distribution of the risk factors.

When risk factors are coded +/−1/2 in a linear model (chosen because SRD is here the effect size):

$$\begin{array}{l} \text{Main}\;\text{Effect}\;\text{of}\;\text{RF}1:[{\text{S}}_{10}(\text{T})-{\text{S}}_{11}(\text{T})+{\text{S}}_{00}(\text{T})-{\text{S}}_{01}(\text{T})]/2\\ \text{Main}\;\text{Effect}\;\text{of}\;\text{RF}2:[{\text{S}}_{01}(\text{T})-{\text{S}}_{11}(\text{T})+{\text{S}}_{00}(\text{T})-{\text{S}}_{10}(\text{T})]/2\\ \text{Interactive}\;\text{Effect}:[{\text{S}}_{10}(\text{T})-{\text{S}}_{11}(\text{T})-{\text{S}}_{00}(\text{T})+{\text{S}}_{01}(\text{T})] \end{array}$$

If there is no *temporal* precedence for RF_1_ and RF_2_, then there are three possible roles for RF_1_ and RF_2_ for the incidence in question:

RF_1_ and RF_2_ are “independent risk factors” if ρ = 0, and if both matter, *i.e.*, INT is non-zero or both ME_1_ and ME_2_ are non-zero. In such cases, the two risk factors play a joint role in determining the outcome and would continue to be of parallel interest. For many disorders, gender and ethnicity are independent risk factors.

One of RF_1_ or RF_2_ “is proxy to” the other if ρ is unequal to zero (RF_1_ and RF_2_ are correlated risk factors), and only one matters. Thus if ME_1_ = INT = 0, then RF_1_ is proxy to RF_2_. If ME_2_ = INT = 0, then RF_2_ is proxy to RF_1_. In such cases, the proxy variable should be set aside from further consideration. For example, a measure of family income is often proxy to a well-measured socio-economic index for the family, because family income is usually one component of the index, but less reliably measured than the index as a whole.

RF_1_ and RF_2_ are overlapping risk factors if ρ is unequal to zero, and both matter. Thus if any two of ME_1_, ME_2_ and INT are not equal to zero, RF_1_ and RF_2_ are overlapping. This situation often arises when two risk factors tap the same underlying construct with about equal, but less than perfect, reliability/validity. Then it is preferable to combine the two risk factors to generate a more reliable/valid measure of whatever their common construct. Combining two somewhat unreliable measures of the same construct would disattenuate reliability and thus increase the effect size. Moreover, to do so might focus attention more precisely on the appropriate underlying causal factor. For example, infant birth-weight and gestational age tend to be highly correlated and risk factors for many of the same subsequent outcomes. Some measure of birth maturity that included consideration of both, perhaps even including other indicators of physiological and neurological maturity at birth, might serve prediction and prevention purposes better than either separately.

On the other hand, when there is temporal precedence, with RF_1_ preceding RF_2_ in time, there are four possibilities:

RF_1_ moderates RF_2_ if ρ = 0 and INT is non-zero. Then the conditional effect sizes for RF_2_ differ depending on whether RF_1_ = 1 or = 0. Since a later risk factor, RF_2_, “works differently” depending on what earlier RF_1_ is, this suggests that the population should be stratified on RF_1_ for further studies. For example, a genotype may be a susceptibility factor for a later environmental risk factor. For those with one genotype, RF_2_ may be a strong risk factor for outcome; otherwise, RF_2_ may be a much weaker risk factor, may not be a risk factor at all, or may even be a protective factor. Indeed, seeking genetic moderators of drug on therapeutic response is the basis for current interest in pharmacogenetics. Moderators are also the basis of personalized medicine[50–53]

RF_2_ mediates RF_1_ if ρ is non-zero, and either INT or ME_2_ is non-zero. In this case, RF_2_ explains part of the effect of earlier RF_1_ on the outcome. When a mediator is identified, this suggests the possibility of a chain leading from RF_1_ through RF_2_ to the outcome. For example, unsafe sex practices lead to HIV infection that leads to onset of AIDS: HIV infection mediates the effect of unsafe sex practices on AIDS. Mediator relationships are important in that the chain provides multiple opportunities for preventive intervention: one might break the chain by breaking any of the links in the chain.

RF_2_ is proxy to RF_1_ if ρ is non-zero, and if MS_2_ and INT are both zero. As is the case for proxy risk factors in absence of temporal precedence, the proxy factor should be set aside. Gender, for example is a risk factor for teen onset of depression. There are many correlates of gender during the pre-teen years, e.g., ball-throwing ability at age 10, that might be found to be risk factors for teen-onset depression when considered individually, but would be found proxy to gender when both were considered. It is probably not worthwhile to teach young girls to throw a ball better to prevent teen depression.

RF_1_ and RF_2_ are independent risk factors if ρ = 0, but INT = 0, *i.e.*, RF_1_ does not moderate RF_2_. As is the case for independent risk factors in absence of temporal precedence, both factors would continue to be of parallel interest.

It should be noted that these definitions are more precise than certain current usages in epidemiology. For example, “independent risk factors” in the MacArthur model are required to be stochastically independent of each other. Usual usage of the term does not require such independence. In the way the term is often used, only proxy risk factors would not be labeled independent risk factors, given large enough sample sizes.

The Last[1] definitions of “mediator” (or “mediating variable” or “intermediate variable”) and of “moderator variable” (or “qualifier variable”) require that the causal pathway from the independent (risk factor of interest) to the dependent variable (onset) be known. Such information is generally not available to a cohort study. Here the issues are, first, temporal precedence and correlation and then the joint association of two risk factors with the outcome. Causality is neither assumed nor inferred.

Moreover the term “confounder” is avoided. Last defines the term as “A variable that can cause or prevent the outcome of interest, is not an intermediate variable (mediator), and is associated with the factor under investigation.” (Page 35). That would preclude mediators explicitly, and preclude moderators and independent risk factors because they are not associated with the factor under investigation, leaving proxies or overlapping factors. However, in practice, the term “confounder” is often used more loosely to refer to risk factors in which the researcher is not specifically interested. Thus in a study examining the relationship of diet and exercise to onset of obesity, a dietician might designate exercise as a “confounder”, while an exercise physiologist might designate diet as a “confounder”.

There is as yet little history of seeking moderating/mediating relationships between risk factors for specific outcomes. Consequently, how to conduct such a search to general moderator/mediator hypothesis, and how to conduct studies to formally test such hypotheses, is still work in progress. However, there are a few examples in the literature that might suggest possible options [54–60].

## 5. Discussion

Many of the problems here discussed are long and well-known, and yet continue to occur. For example, Caspi and colleagues [57] reported in 2003 on the moderating effect of a gene (5-HTT) on an environmental risk factor (stress) on major depression (DSM-IV definition) in a prospective community cohort followed from birth to 26 years. Their finding was particularly interesting since it suggests a genetic moderator of an environmental effect on a disorder. Risch *et al.* in 2009 [60] evaluated whether that finding had since been confirmed or refuted. However, of the 13 studies they reported as attempts to replicate, 8 were cross-sectional studies, that could legitimately evaluate neither stress as a risk factor for major depression, nor any moderator hypothesis. Of the five remaining prospective cohort studies, none covered the same age range as did the Caspi *et al.* study, with two sampling those 65 years of age or older. No effect sizes other than Odds Ratios were reported for any of the studies. Thus, none of the reported studies actually evaluated the same effect as did the Caspi *et al.* study, largely because issues related to time were ignored.

Science progresses by identifying its weaknesses and repairing them. However, it is always very hard to give up methods long used in previous studies and thus very familiar, to be replaced with new and unfamiliar methods. “That’s not the way it is always done, or the way everyone does it!” is a common rejoinder to suggestions for alternative approaches to deal with the problems here discussed.

Such resistance is particularly and predictably strong when the alternative approaches are more difficult and costly to implement, which is here true. To be asked to stratify a mixed age sample, say 20–80 years of age, into relatively short age strata (say entry at 20–24, 25–29, *etc.*), and to do analysis separately within each age stratum, results in a reduction of sample size and thus power to detect effects, inevitably an unwelcome suggestion. However, the alternative is the risk of detection of false positive results.

Resistance is particularly, and again, understandably, strong when alternative methods are *less* likely to produce “significant” results, and *more* likely to indicate that the effect of any single risk factor is quite weak, because such alternative methods counter the tendency of incorrect methods to produce false positive results. This may be one of the major reasons why Odds Ratio has been so hard to displace as an effect size: it tends to exaggerate what are often trivial associations and results in publications of results that only later are shown to be exaggerations at best, and at worst, false positive results.

The rejoinder hardest to refute is that the effect of time can be dealt with simply by “adjusting for time” in a linear model. In some cases this may indeed be possible. However, the assumptions and fit of such models should be carefully checked, for if the assumptions that underlie valid results from application of such models do not hold in the population sampled, the results of such adjustments may be more biased than the results in absence of adjustment.

In summary, the issue of time should be central to all thinking in epidemiology research, which would necessitate careful thinking about sampling, measurement, design, analysis, and, perhaps most important, about the interpretation of the results from such studies that might influence clinical decision-making and subsequent clinical research.

## Figures and Tables

**Table 1.** The joint distribution of two binary risk factors (RF1 and RF2). with marginal probabilities P = Prob(RF2 = 1) and Q = Prob(RF1 = 1), and the product moment correlation coefficient (ρ) between them.

	RF1 = 1	RF1 = 0	
RF2 = 1	PQ+ρ(PP’QQ’)^1/2^	PQ’−ρ(PP’QQ’)^1/2^	P
RF2 = 0	P’Q−ρ(PP’QQ’)^1/2^	P’Q’+ ρ(PP’QQ’)^1/2^	P’ = 1 − P
	Q	Q’ = 1 − Q	

**Table 2.** The incidence of disorder by time T for each combination of RF1 and RF2, and the marginal SRDs.

	RF1 = 1	RF1 = 0	Marginal SRD for RF1
RF2 = 1	1 − S_11_(T)	1 − S_10_(T)	S_10_(T)−S_11_(T)
RF2 = 0	1 − S_01_(T)	1 − S_00_(T)	S_00_(T)−S_01_(T)
Marginal SRD’s for RF2	S_01_(T)−S_11_(T)	S_00_(T)−S_10_(T)	
